# Methods of Attenuating Ischemia-Reperfusion Injury in Liver Transplantation for Hepatocellular Carcinoma

**DOI:** 10.3390/ijms22158229

**Published:** 2021-07-30

**Authors:** Łukasz Masior, Michał Grąt

**Affiliations:** 1 Department of General, Vascular and Oncological Surgery, Medical University of Warsaw, Stępińska Street 19/25, 00-739 Warsaw, Poland; 2 Department of General, Transplant and Liver Surgery, Medical University of Warsaw, Banacha Street 1A, 02-097 Warsaw, Poland; michal.grat@gmail.com

**Keywords:** ischemia-reperfusion injury, liver transplantation, hepatocellular carcinoma, preconditioning, machine perfusion

## Abstract

Hepatocellular carcinoma (HCC) is one of the most frequent indications for liver transplantation. However, the transplantation is ultimately associated with the occurrence of ischemia-reperfusion injury (IRI). It affects not only the function of the graft but also significantly worsens the oncological results. Various methods have been used so far to manage IRI. These include the non-invasive approach (pharmacotherapy) and more advanced options encompassing various types of liver conditioning and machine perfusion. Strategies aimed at shortening ischemic times and better organ allocation pathways are still under development as well. This article presents the mechanisms responsible for IRI, its impact on treatment outcomes, and strategies to mitigate it. An extensive review of the relevant literature using MEDLINE (PubMed) and Scopus databases until September 2020 was conducted. Only full-text articles written in English were included. The following search terms were used: “ischemia reperfusion injury”, “liver transplantation”, “hepatocellular carcinoma”, “preconditioning”, “machine perfusion”.

## 1. Introduction

Liver transplantation is currently the most effective form of therapy for hepatocellular carcinoma (HCC), which usually develops in cirrhotic liver [1]. Each transplantation is associated with ischemia-reperfusion injury (IRI). It results from the necessity to store the organ until it is implanted in the recipient and the blood flow is restored. During the procedure, the graft is exposed to periods of warm and cold ischemia. Cold ischemia time (CIT) starts from the beginning of the donor’s liver flushing with cold preservation fluid until the graft is removed from the ice prior to implantation. Afterwards, a period of warm ischemia begins (WIT, warm ischemia time) and continues until graft reperfusion. In the case of donation after cardiac death (DCD), during organ procurement the donor’s warm ischemia is also measured, which is defined as the period from the donor’s cardiac arrest until the liver is flushed with a cold preservation fluid. Functional WIT (fWIT) is a period that starts when the donor’s mean arterial blood pressure (MABP) drops to ≤45 mmHg [2]. IRI is a factor that adversely affects both the function of the graft and its survival. A similar negative effect is observed in patients undergoing kidney transplantation [3]. Moreover, numerous studies have proved that IRI is a factor promoting cancer progression [4,5,6]. It is even more important as currently the organs obtained from extended criteria donors (ECDs) are used routinely. These organs, in turn, are more susceptible to injury [7]. Therefore, understanding the mechanisms and methods of reducing the effects of this process is of key importance and may positively affect outcomes of liver transplantation in HCC patients.

## 2. Mechanism Responsible for Ischemia-Reperfusion Injury

**Figure 1:**
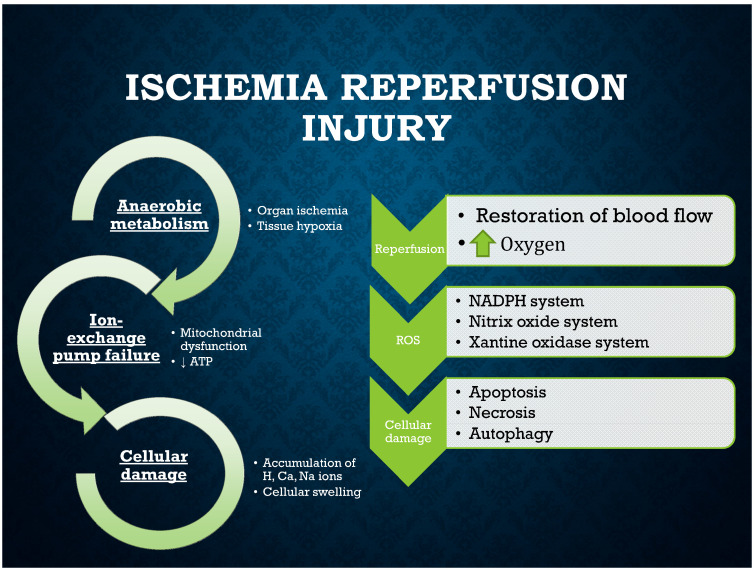
Mechanism responsible for IRI development.

The damage consists of two phases: tissue ischemia of various durations and damage related to the restoration of blood flow (Figure 1). In the first phase, tissue hypoxia induces anaerobic metabolism. This results in mitochondrial dysfunction and a decrease in adenosine triphosphate (ATP) production. In turn, lower production of ATP results in dysfunction of the pumps transporting ions inside and outside the cells. This causes the accumulation of hydrogen, sodium, and calcium ions inside the cells, which leads to cellular swelling. In addition, the cellular pH is lowered, which results in damage to nuclear chromatin and broadly disturbs the function of enzymes [8,9]. After the blood flow has been restored to supply oxygen, there is a second phase of cellular injury that results from the production of oxygen free radicals related to nicotinamide adenine dinucleotide phosphate (NADPH), xanthine oxidase, and nitric oxide. Oxidative stress causes cell death by the mechanisms of apoptosis, necrosis, and autophagy [8,9,10]. Necroptosis, one of the other types of programmed cell death, also plays a role [11].

## 3. Liver Damage following Ischemia-Reperfusion Injury

IRI in the liver has two phases. In the early period, the activity of the complement complex, Toll-like receptors (TLR) and interleukin 23 (IL-23), induced by oxidative stress, activates Kupffer cells. These cells, together with CD-4 lymphocytes, by the production of pro-inflammatory cytokines, are responsible for the influx of neutrophils, which are responsible for liver damage [12,13]. Among the numerous cytokines involved in IRI, tumor necrosis factor alpha (TNF-alpha) seems to play a key role [14]. Different cells are predominantly affected during cold and warm ischemia. In the course of warm ischemia, mainly hepatocytes are injured. Cold ischemia predominantly affects endothelial cells (LSEc-liver sinusoidal endothelial cells) [15,16]. Damage to these cells is responsible for vasoconstriction modulated mainly by the production of thromboxane A2. Another element is the activation of platelets and the formation of microclots, which worsens the circulation in the hepatic sinuses and deepens the organ ischemia [15]. An important role of the IRI mechanism is played by intracellular protein complexes, inflammasomes, which are responsible for the coordination of the inflammatory response [17]. In addition, microRNAs (miRNAs) and HIFs (hypoxia-inducible factors) are the next ones whose role is being more and more studied [18]. In addition, authors from China identified specific genes and proteins with a potentially key role in the course of IRI. These include ATF3, CCL4, DNAJB1, DUSP5, JUND, KLF6, NFKBIA, PLAUR, PPP1R15A, and TNFAIP3. They belong to the group of differentially expressed genes (DEGs). Moreover, ten genes/proteins (HBB, HBG2, CA1, SLC4A1, PLIN2, JUNB, HBA1, MMP9, SLC2A1, and PADI4) were also revealed. Their close cooperation seems to be an important contributor to IRI during liver transplantation [19]. As mentioned before, livers from extended criteria donors are more exposed to IRI. The worse function of organs from older donors is related to impaired autophagy mechanisms, which play a protective role for hepatocytes [20]. Increased risk is also observed in organs with steatosis and from DCD donors. In steatotic livers, the production of free oxygen radicals increases as a result of lipid peroxidation. Another mechanism is related to microcirculation disturbances [11,18]. In DCD organs, the greater susceptibility to IRI is mainly related to the long duration of warm ischemia time [21].

## 4. Role of Ischemia-Reperfusion Injury in Cancer Growth Promotion

IRI has a direct impact not only on the function of the transplanted liver, but it also creates an environment promoting implantation and growth of cancer cells. In experimental animal studies, liver IRI has been shown to increase the mobilization of endothelial progenitor cells (EPCs) from the bone marrow, which stimulate angiogenesis. This in turn translates into a faster growth of the liver tumors [22,23]. The CXCL10/CXCR3 chemokines play an important role in this process. These chemokines induce mobilization of regulatory T-cells (Treg) that, by inhibiting the immune response, may promote the progression of hepatocellular carcinoma [24]. In a study by van der Bilt et al., inducing liver ischemia accelerated several times the growth of cancer cells in the ischemic parenchyma [5]. This phenomenon is especially visible around the areas of liver necrosis rich in inflammatory cells and apoptotic hepatocytes. Growth stimulation is associated with increased expression of HIF-1α as a response to tissue hypoxia. HIF-1α has proliferative and angiogenesis-stimulating activities [25]. An important element is IRI-induced increased VEGF (vascular endothelial growth factor) and MMX-9 metalloproteinase expression. The role of metalloproteinases in cancer progression is complex and includes stimulating angiogenesis and the ability to invade the stroma by cancer cells [26,27,28]. In an experimental model in rats, IRI was also shown to be associated with faster growth of hepatocellular carcinoma cells. It was modulated by increased expression of HIF-1α, as well as activation of the IL-6-JAK-STAT3 signaling pathway [29]. A similar effect on the progression of hepatocellular carcinoma was observed by authors from Hong Kong who tested the animal model of transplantation and acute small for size syndrome [30]. The graft damage resulted in faster growth of the tumor and its greater invasiveness. The mechanism responsible for this includes creating a favorable microenvironment, activation of specific signaling pathways, and increased cell proliferation, which was assessed by Ki-67 expression. Orci et al. came to similar conclusions by studying the IRI in mice livers with steatosis. IRI resulted in a faster growth of HCC cells in markedly steatotic livers. Moreover, the tumor burden was similar in both ischemic and non-ischemic parts of the organ. It suggests a proliferative effect on tissues that are not directly affected by IRI [31].

## 5. Ischemia Reperfusion Injury and HCC Prognosis

Insight in the role of IRI in HCC progression provided by the results of experimental studies, for obvious reasons, resulted in evaluation of its relevance in patients with HCC, treated both by liver resection and transplantation. During liver resection, IRI is most often associated with the use of temporary closure of the hepatoduodenal ligament (Pringle maneuver). Analysis of the two RCTs (randomized controlled trials) by Lee at al. showed that the use of intermittent Pringle maneuver (IPM) may even prolong overall survival, paradoxically in the group of patients with cirrhosis. There was no difference in recurrence-free survival. However, the authors admit that the mechanism of this phenomenon is still not well understood [32]. In another study by Xia et al. the effect of temporary closure of blood inflow to the liver on both long-term and recurrence-free survival in patients with HCC undergoing liver resection was not observed [33]. Another study investigated the effect of IRI on the results of liver transplantation in HCC patients. A total of 195 patients treated between 2001 and 2016 were analyzed. The severity of IRI was assessed by the asparagine transaminase (AST) and lactate dehydrogenase (LDH) activity after reperfusion. The authors showed that recurrence-free survival was significantly shorter in patients with AST ≥ 1896 U/L and LDH ≥ 4670 U/L. The adverse effect of IRI was observed mainly in the group of patients meeting the Milan criteria, and to a lesser extent in the group meeting the Up-to-7 criteria. No negative effect was found in patients with more advanced disease. These important findings suggest the possibility of using marginal grafts in recipients with greater tumor burden, thus offering liver transplantation to a wider group of patients. Moreover, strategies to minimize IRI seem to be of less importance in this particular cohort [6]. Surgeons from Germany also noticed worse results related to IRI in patients with HCC undergoing orthotopic liver transplantation. In their group of 103 patients, long-term results were significantly impaired in patients with WIT longer than 50 min. Three-year disease-free survival was 92.8% and 42.0% for patients with WIT ≤ 50 min and > 50 min, respectively. The influence of WIT was particularly significant in the group of patients with higher risk of recurrence estimated on the basis of preoperative PET (position emission tomography) scan (PET-avid patients). In this group, WIT > 50 min was found to be the only risk factor in the multivariable analysis, which translated into poor 3-year recurrence-free survival of only 18.8% compared to 80% among patients with WIT ≤ 50 min. In the multivariable analysis, risk factors associated with recurrence, apart from WIT > 50 min, were PET-positive patients, alpha-fetoprotein (AFP) concentration > 400 IU/mL, and tumors outside Milan criteria [34]. Similarly, Nagai et al., analyzing the results of liver transplantation in 391 HCC patients, showed an adverse effect of the length of ischemic times. In the whole group both CIT > 10 h and WIT > 50 min were independent risk factors increasing the risk of HCC recurrence in the multivariable analysis. Other significant risk factors were tumors exceeding Milan criteria, AFP concentration > 200 ng/mL, micro and macrovascular invasion, and poor tumor differentiation. When patients were stratified by presence of vascular invasion (VI), ischemic times (WIT and CIT) persisted as risk factors in a subgroup of recipients with VI, conversely to those without VI where no impact was noticed [35]. As mentioned previously, organs from extended criteria donors are at increased risk of more severe IRI. An analysis of the SRTR (Scientific Registry of Transplant Recipients) database, including nearly 10,000 patients undergoing liver transplantation due to HCC between 2004 and 2011, revealed an adverse effect of donor-related parameters on HCC recurrence. Factors such as BMI (body mass index) ≥ 35 kg/m^2^, age > 60 years, diabetes, liver steatosis > 60%, and WIT in a DCD donor > 19 min were associated with a higher risk of recurrence. All the analyzed factors increase the liver’s susceptibility to IRI, which has been shown to stimulate tumor progression [36]. Another analysis of the data of 29,020 SRTR patients transplanted for both HCC and benign diseases focused on analyzing the impact of donor-related factors on treatment outcomes. Graft quality was assessed by the DRI (Donor Risk Index). The results showed a similar influence of donor-related and recipient factors on the prognosis of HCC patients [37].

It should be noted that the use of suboptimal grafts for transplantation in HCC patients is not always associated with worsening of the results. A group from King’s College Hospital in London analyzed data of 347 patients, 91 (26.2%) of whom underwent DCD transplants. The long-term outcomes did not differ between DCD and Death Brain Donors (DBD). Five-year overall survival was 80% and 72.4% for DBD and DCD donors, respectively (*p* = 0.115). With regard to cancer-specific survival, similarly there was no significant difference between the DBD and DCD group at 5 years (88.5% vs. 87.3%; *p* = 0.700). Donor type was not found to be a risk factor worsening survival and increasing a risk of HCC recurrence in both univariate and multivariable analyses. However, the authors emphasized that the DCD organs were of good quality. It seems that the center’s experience was also an important factor, which translated into short WIT and CIT [38]. The favorable results in a selected group of HCC patients transplanted with DCD grafts was also confirmed by other high-volume centers. Silverstein et al. elucidated the role of transplantation using DCD organs in 7563 patients with HCC who underwent operations in the US between April 2012 and December 2016. A total of 7.5% of recipients were transplanted with DCD grafts, and 3-year recurrence rates were 7.6% and 6.4% for DCD and DBD donors, respectively (*p* = 0.67). Overall survival after DCD transplantation was significantly impaired only in certain cohorts such as those with AFP > 100 ng/mL, Risk Estimation of Tumor Recurrence after Transplant (RETREAT) score ≥ 4, and in recipients with contrast-enhanced multiple liver lesions found in radiological studies directly before surgery [39].

## 6. Strategies to Attenuate Ischemia-Reperfusion Injury

The unfavorable consequences of IRI resulted in the search for a strategy to diminish its impact. Taking into account the complex mechanism of this phenomenon, researchers tested various pharmacological interventions as well as invasive procedures that could potentially modify injury, limiting its adverse effect. A summary of these methods is presented in Table 1 and Table 2.

## 7. Pharmacological Strategies

Numerous studies have focused on the potential role of anesthetic drugs in reducing IRI-related liver damage. In an experimental study in an animal model, Xu et al. demonstrated the beneficial effect of the use of propofol and sevoflurane on the reduction in ischemia-reperfusion damage. The positive effect was associated with their anti-apoptotic role as well as changes in the production of inflammatory cytokines (TNF-α, Il-1, Il-6, Il-10) and reduced generation of oxygen free radicals in liver tissue [48]. The attenuating effect of propofol was also confirmed by other in vitro experiments [49]. Other substances were also assessed for their impact on IRI modulation. Researchers from China showed a beneficial effect of remifentanil. An experimental rat model was used to conduct the study. As a major mechanism of action, authors suggest suppression of cellular apoptosis [50]. Sufentanil was also proven to have a protective effect according to the study of Lian et al. The mechanism responsible for its activity is based mainly on reducing the production of pro-inflammatory cytokines (HIF-1a, TNF-a, IL-1b, and IL-6), with less severe IRI reflected by lower AST and LDH activity [51]. Edaravone is a drug approved in the US and Japan to treat neurological diseases. This substance belongs to a group of scavengers of oxygen free radicals. Based on the key role of reactive oxygen species in the IRI pathophysiology, Abe et al. investigated effect of Edaravone on the course of liver IRI in rats. Authors used in vitro and in vivo models. In both experiments, attenuating potential of Edaravone was noticed as measured by AST activity, phosphatidylcholine hydroperoxide (PCOOH) concentration, and changes in cell energy charge [52]. The benefits were also confirmed in further experiments [53].

Human clinical trials of anesthetic drugs were conducted in patients undergoing liver resection. The center from Zurich in a prospective randomized trial evaluated preconditioning with sevoflurane in patients undergoing liver resection with the simultaneous use of the Pringle maneuver [54]. Sixty-four patients were included in the study. Significantly lower postoperative transaminases activity was observed in the sevoflurane group, reflecting less damage to the liver tissue. Preconditioning was also significantly associated with reduction in the number of all complications, including severe ones (IIIb-V according to the Clavien–Dindo classification). The same group of researchers conducted another randomized study, assessing so-called postconditioning, i.e., the use of sevoflurane during reperfusion after releasing of the temporary Pringle maneuver [55]. The hepatoprotective effect of the tested drug was also demonstrated. A systematic review by Abu-Amar et al. analyzed 18 RCTs in which 17 different substances with a potentially liver-protective effect during resection under control ischemia were assessed. Four of them, namely methylprednisolone, trimetazidine, dextrose, and ulinastatin, showed a beneficial effect. However, authors concluded that the data are not robust enough to recommend their routine use [56]. Unfortunately, data assessing the role of the above-mentioned substances in patients undergoing liver transplantation, especially for HCC, are limited. However, taking into account their potentially beneficial hepatoprotective effect, it seems reasonable to test them in liver transplant patients, preferably in the context of prospective randomized trials.

N-acetylcysteine (NAC) is a drug that has an established role in the treatment of patients with acetaminophen poisoning. In an experimental study on rabbits, liver ischemia was induced by inflow occlusion for 60 min followed by 7 h of reperfusion. NAC was administered 15 min before reperfusion followed by continuous infusion during the whole reperfusion period. A beneficial effect has been shown on the reduction in IRI assessed by lower post-reperfusion alanine transaminase (ALT) activity and an improvement in indocyanine green clearance [57]. The potential role of N-acetylcysteine has also been confirmed in patients undergoing liver transplantation. In the prospective randomized trial of Santiago et al., 25 out of 50 transplanted patients received NAC, starting from the anhepatic phase, and continued intravenous infusion for 24 h. The effect of NAC was determined by the concentration of IL-4 and IL-10 in peripheral blood samples. The use of NAC was associated with significantly higher concentrations of both anti-inflammatory cytokines before (IL-4, IL-10) and after reperfusion (IL-4), which may have an attenuating effect on IRI [40].

Another drug evaluated in humans was the antibiotic Rifaximin. Researchers from UCLA (University of California, Los Angeles) analyzed a group of 206 liver transplant patients who received Rifaximin preoperatively [41]. Both in the whole study group and after the propensity score matching, it was shown that the use of Rifaximin ≥ 28 days was associated with significantly lower postoperative aminotransferases activity and a significantly lower frequency of EAD (early allograft dysfunction; 10.3% vs. 33.3%). As a mechanism of action, the authors mention the reduction in the inflammatory response caused by decreasing the activity of neutrophils and macrophages. Prostaglandin E1 is another substance showing beneficial effects in patients undergoing liver transplantation. Its analogue (Alprostadil) was used in patients after liver transplantation due to HCC [42]. The study included a group of 106 patients. The 5-year recurrence-free survival was 85.7% and 63.1% for patients treated with Alprostadil and those who did not receive it, respectively. The beneficial mechanism of action is related to the attenuation of IRI that, as mentioned earlier, promotes the progression of hepatocellular carcinoma. However, the retrospective nature of the study does not allow certain conclusions to be drawn regarding the routine use of this drug after transplantation. Early graft function may be improved also by probiotics. In the randomized controlled study including 55 patients, continuous probiotics administration before liver transplantation (26 patients) was associated with faster normalization of AST and ALT activity as well as lower bilirubin concentration in the early post-transplant period [58].

## 8. Mechanical Strategies

One of the well-known methods that has a protective effect on the liver is ischemic preconditioning (IP). IP consists of short period(s) of inflow occlusion followed by reperfusion of the liver. It serves as a preparation before the subsequent major liver damage. In a study by Rodriguez-Reynoso et al., rats were subjected to various IP lengths (5, 10, 20 min + 10 min of reperfusion) followed by a long 90 min ischemia [59]. Optimal results were obtained with 10 min IP. IP was associated with a decrease in the magnitude of the IRI as expressed by increased production of nitric oxide, decreased production of oxygen free radicals and pro-inflammatory cytokines (TNF-α, IL-1), with accompanying less neutrophil infiltration of the liver. The 7-day survival was also the longest after IP lasting 10 min. The 20 min IP was associated with a significantly increased IRI, which translated into 100% mortality of tested animals. Experimental studies have shown a beneficial effect of IP not only against IRI but also revealed the inhibitory effect on HCC progression. Authors from Switzerland proved that applying IP in livers with steatosis resulted in a significant decrease in tumor volume compared to organs exposed directly to IRI. Importantly, tumor load was similar in livers protected by IP and those not affected by ischemic injury [31]. Animal studies also show the beneficial effects of remote ischemic preconditioning (RIP). This concept is based on the induction of short-term periods of ischemia and reperfusion in a specific organ, which has a protective effect on distant organs. Korean researchers conducted a trial using a rat model [60]. RIP was established by occluding the femoral vessels. An attenuation of IRI in the liver was observed, mainly through the reduction in oxidative stress. The combination of IP and RIP enhances the beneficial protective effect, as confirmed in a study by Li et al. [61]. In an experiment on mice, the combination of both methods was associated with the greatest protection, expressed by lower transaminase activity, lower production of pro-inflammatory cytokines, reduction in oxidative stress, and increased anti-apoptotic effect. Additionally, induction of ischemia and subsequent reperfusion of the liver prior to its harvest for transplantation (pre-retrieval reperfusion) may be a valuable strategy. Oldani et al. evaluated this technique in transplanted rats that were additionally implanted with HCC cells via the portal vein [62]. Livers were removed immediately after the ischemic period or after ischemia followed by a 2 h reperfusion. In the group of livers transplanted after the ischemia/reperfusion, the magnitude of IRI and the growth of HCC were less pronounced. The mechanism behind this consists of a reduction in the inflammatory response (lower IL-1 and higher IL-10 concentration) and a decrease in the expression of genes stimulating the growth of HCC cells (Hmox1, Hif1a, and Serpine1).

Research on IP and RIP was also carried out in patients undergoing liver transplantation. In a prospective randomized study by Amador et al., the effect of a 10 min Pringle maneuver followed by a 10 min reperfusion in donors was investigated [63]. The study included a group of 60 donors, and the intervention was performed in half of them. The use of IP was associated with a significant attenuation of IRI as measured by lower AST activity in the early postoperative period. The frequency of reoperations and primary non-function (PNF) was also lower in the IP group. A positive effect in the context of IRI was observed in another study from Italy [43]. IP was used in 23 donors (10 min of ischemia + 15 min of reperfusion) and compared to a group of 24 standard donations. Postoperative aminotransferases activity was lower in the IP group, but the function and survival of the graft did not differ significantly between groups. Interesting conclusions can be found in the work of Koneru et al. [64]. In this prospective randomized study, which included a group of 101 patients transplanted from deceased donors, 50 donors underwent 10 min ischemia of the liver followed by reperfusion until the start of liver flushing with a cold preservation fluid. There was an increase in IRI in the postoperative course in the IP group, which was determined by higher activity of transaminases. Surprisingly, higher levels of anti-inflammatory IL-10 and a lower risk of early rejection were also observed. A meta-analysis of 10 studies on IP in patients undergoing liver transplantation showed that IP was associated with a statistically significantly lower AST activity on the third postoperative day. Statistically insignificant lower 1-year mortality and lower rates of PNF were also found [65]. A study to evaluate RIP in patients undergoing liver transplantation was conducted by researchers from London (RIPCOLT trial) [44]. The study group consisted of 40 patients. RIP was used in 20 recipients, achieved by lower limb ischemia and included three cycles (5 min of ischemia + 5 min of reperfusion) prior to skin incision. During the early post-transplant period (90 days) no significant benefits of the intervention were observed. According to the authors, a longer period of observation is necessary as well as modification of the study protocol, possibly with an extension of the limb ischemia time. Another randomized study to evaluate RIP was conducted in Korea in a group of patients undergoing liver transplantation from living donors [66]. RIP was induced in donors by upper limb ischemia (5 min of ischemia + 5 min of reperfusion). No beneficial effect was observed in donors, while the use of RIP had a protective effect in recipients as assessed by lower post-operative AST activity.

For many years, intensive research has been carried out on how to improve the outcomes of liver transplantation using the so-called machine perfusion. Its role is to maintain/improve the function of the graft before the liver is implanted and the physiological blood flow is restored. This is done by perfusing the liver with oxygenated perfusion solution or blood, possibly with the addition of specific drugs and nutrients. Perfusion can be performed by both hypothermic (hypothermic oxygenated perfusion, HOPE) and normothermic (normothermic machine perfusion, NMP) approaches and their combination [67]. Friend et al. conducted a NMP evaluation study using human livers disqualified from transplantation [68]. Perfusion lasting up to 24 h has been shown to be technically possible and safe. The authors emphasize a number of benefits, such as the possibility of improving and assessing the function of the graft before transplantation or extending the time frame in which the procedure is possible, which is of key importance for logistics reasons. The continuous development of technology allowed for the construction of devices allowing for perfusion lasting up to 7 days in experimental environments [69]. Such a long period of time allows even the observation of the process of liver regeneration with all significant consequences. A study by Schlegel et al. demonstrated the effectiveness of the HOPE technique in an experiment on rats subjected to liver transplantation in the DCD model [70]. Ten organs were preserved with a static cold storage (SCS), while 10 livers were perfused for 1 h before transplantation. The severity of IRI was markedly lower in the perfused livers. This was reflected in significantly lower transaminases activity as well as higher values of the Quick index and coagulation factor V 12 h after transplantation. Importantly, a protective effect against biliary injury was also demonstrated. It is a crucial finding as increased risk of cholangiopathy is one of the major drawbacks of transplantation from DCD donors. Studies with the use of NMP confirmed the effectiveness of this technique. A team from the Cleveland Clinic, in an experiment with pig livers, compared 15 organs treated with NMP with classic SCS [71]. In addition to the safety of the method, a significantly less pronounced IRI was shown in livers after NMP, expressed by lower transaminases and LDH activity and increased bile production. Moreover, in the histopathological examination, hepatocyte necrosis was more severe in livers preserved with SCS. Another interesting trial was published by Oldani et al. An experimental rodent model of liver transplantation with DCD grafts was established to conduct the study. Before transplantation, grafts were perfused for 2 h using both normo and hypothermic techniques. Controls were DCD non-perfused grafts and fresh livers. Normothermic perfusion translated into a better postoperative aminotransferase profile compared to transplantation using DCD livers and DCD grafts after hypothermic perfusion. However, neither hypothermic nor normothermic perfusion showed benefits in terms of HCC growth [72].

Clinical trials have demonstrated the superiority of the HOPE technique over the classic SCS preservation in patients transplanted from DCD donors. In a study by Dutkowski et al., peak transaminases, ischemic cholangiopathy, and biliary complications occurred significantly less frequently in patients whose grafts were perfused before implantation [45]. Importantly, the 1-year survival of the graft was also better (90% vs. 69%). The use of the HOPE technique in DCD donors provides comparable results of transplantation to those where DBD organs were used [73]. The first randomized trial comparing NMP and the classic method of preservation was published in 2018 [46]. In a multicenter study 220 patients who had livers procured from DBD or DCD donors were analyzed. The organs were randomly assigned to the control (SCS) or experimental group (NMP). In the NMP group, EAD was much less frequent (OR 0.263; 95% CI 0.126–0.550; *p* < 0.001), which was reflected in the 50% reduction in postoperative AST peak. In addition, non-anastomotic biliary strictures were less frequent in the DCD group (NMP 11.1% vs. SCS 26.3%; *p* = 0.180). Importantly, the percentage of discarded organs was statistically higher in the SCS group (24.1% vs. 11.7%, *p* = 0.008). Impact of machine perfusion on HCC outcomes after liver transplantation was assessed by a team from Zurich [74]. A total of 140 patients (70 in each group) were analyzed. Recurrences occurred in 5.7% of patients who received organs from DCD donors that were perfused using HOPE, and in 25.7% of those transplanted with organs from DBD donors (no perfusion). The 5-year recurrence-free survival was also better (93% vs. 72%; *p* = 0.027). The superiority of HOPE was confirmed by external validation comparing these results with the results obtained in patients transplanted in Birmingham. Five-year recurrence-free survival in UK patients whose grafts were not perfused was 82.7% and 81.2% for DCD and DBD donors, respectively. An extension of the HOPE technique (in which perfusion is provided through the portal vein) is the so-called dual HOPE (DHOPE). The method involves perfusion of the liver through both the portal vein and the hepatic artery. In a study by van Rijn et al., the results of liver transplantation from DCD donors, 10 of which were perfused with DHOPE, were analyzed [75]. The results were compared to 20 procedures using the classic method of preservation. Intrahepatic ATP levels increased significantly during DHOPE. In the postoperative course, the DHOPE group showed a lower intensity of IRI and a smaller number of biliary complications. The 1-year graft and patient survival were also longer in the perfusion group. Encouraging results were also obtained by combining the hypo and normothermic perfusion [76]. Another strategy offered by machine perfusion is its use in donors after their death is determined and before the organ procurement (regional NMP). A study from France compared regional NMP in DCD donors with transplantations from DBD donors [47]. It was a retrospective matched-control study that included 150 patients. In 50 patients, perfusion was performed. In the NMP group, the aminotransferase activity was significantly lower in the early postoperative period. The incidence of EAD, biliary complications, and 2-year graft and patient survival were comparable in both groups, which proves the effectiveness of this technique and potentially extends the pool of organs that can be used for transplantation. However, attention was drawn to a greater frequency of graft loss due to recurrent HCC in patients from the NMP group (12% vs. 3%; *p* = 0.007). Accordingly, the authors suggest disqualifying patients with more advanced tumors (alpha score > 2) based on the French alpha-fetoprotein model. Very good results with the use of regional NMP in DCD donors were confirmed in a study evaluating 123 perfusions performed in 2015–2018 as part of a national program conducted in France [2]. The studies also showed the superiority of regional NMP comparing to the super rapid recovery (SRR), which is the current standard technique in DCD procurement [77].

## 9. Other Strategies

Given the detrimental impact of extending CIT and WIT, strategies that optimize organ allocation and coordination between liver donation and transplant teams can significantly improve treatment outcomes. Cameron et al., from Los Angeles, analyzed this problem in a group of over 1000 patients treated with liver transplantation [7]. The risk score was created based on the following factors: donor’s age > 55 years, donor stay in hospital > 5 days, CIT > 10 h, and WIT > 40 min. One point was awarded for each parameter. In the case of organs with a score of 0–2, the 1-year patient survival was 88%, 82% and 77%, respectively. For grafts scoring 3 points, 1-year survival was only 48%. The disadvantageous effect of using lower-quality organs was most apparent in recipients requiring urgent transplantation. In the conclusions, the authors emphasize the possibility of obtaining good results, provided that organs and recipients are precisely matched. Although this study did not focus on HCC patients, it highlights the role of precise coordination of entire complex process of transplantation in improving outcomes, with particular emphasis on ischemic times. This allows for obtaining good results also with the use of suboptimal organs that are more susceptible to IRI with it all deleterious effects. Another interesting observation came from animal study regarding preconditioning induced by exercise training [78]. Animals subjected to 4-week training (treadmill running) before induction of liver ischemia were assessed. Additionally, colorectal cancer cells were implanted via portal vein before IRI initiation. Apart from protection against IRI, exercise training decreased number of liver metastases formation three weeks after IRI. These promising results show that it is a very safe and cost-effective strategy. Undoubtedly, the role of exercise training needs to be explored further.

## 10. Conclusions

Ischemia-reperfusion injury is an integral part of the liver transplant procedure. Recent data presented by one of the biggest liver transplant centers in the world clearly show the detrimental role of this phenomenon. In the study of Ito et al., from UCLA, among 506 liver transplant patients IRI was diagnosed in 87.6% cases. Moderate/severe IRI was significantly associated with EAD occurrence (*p* = 0.001). Moreover, increasing IRI severity was found to be a risk factor of a shorter 6-month graft survival (*p* = 0.008) [79]. Similar conclusions were drawn by Bastos-Neves et al., after analyzing 602 liver transplantations. In their analysis grade IV IRI was strongly correlated with EAD development (*p* = 0.002) [80].

Negative impact of IRI on both the function of the graft and the oncological results in patients with HCC naturally prompted researchers to seek methods aiming at control of IRI extent. Many promising substances have been tested in animal models, but most of them did not progress beyond the experimental phase. Some drugs with potentially beneficial effects were evaluated in clinical trials, but their retrospective character does not allow for drawing unambiguous conclusions about their routine use. Ischemic modulation techniques (IP and RIP) need further studies; however, current data are encouraging at present, and the most important strategy seems to be to optimize the overall transplantation process and to use machine perfusion. It might be that the future will belong to transplantations in which the problem of ischemia does not exist at all. Ischemia-free transplantation using continuous NMP has been already described by surgeons from China [81]. Although technical feasibility and satisfactory results were shown, still many problems have yet to be overcome before ischemia and subsequent IRI will no longer apply to patients undergoing liver transplantation.

## Figures and Tables

**Table 1:** Summary of the different strategies employed to mitigate ischemia-reperfusion injury.

	Non-Invasive Strategy	Invasive Strategy
**PHARMACOLOGICAL**		
1. Sevoflurane, propofol 2. Sufentanil 3. Edaravone 4. N-acetylcysteine 5. Rifaximin 6. Prostaglandin A1 7. Others (methylprednisolone, trimetazidine, dextrose, and ulinastatin)	**YES**	**NO**
**MECHANICAL**		
*Hepatic Inflow Modulation* 1. Ischemic preconditioning (IP) 2. Remote ischemic preconditioning (RIP) 3. Pre-retrieval reperfusion	**NO**	**YES**
*Machine perfusion* 1. Hypothermic perfusion (HOPE) 2. Dual hypothermic perfusion (D-HOPE) 3. Normothermic perfusion (NMP) 4. Regional normothermic perfusion	**NO**	**YES**

**Table 2:** Selected studies assessing various mitigation strategies against IRI.

Study	Year	Intervention	Results
Santiago FM et al. [40]	2008	N-acetylcysteine (NAC) administered during liver transplantation (anhepatic phase)	Randomized controlled trial 25 patients in each group (NAC vs. placebo) Higher concentrations of both anti-inflammatory cytokines before (IL-4, IL-10) and after reperfusion (IL-4) in NAC group
Ito T et al. [41]	2019	Rifaximin given before liver transplantation ≥28 days vs. none or <28 days	Retrospective study Propensity score matching (39 patients in each group) Longer use (≥28 days) associated with lower postoperative aminotransferases activity and lower frequency of EAD (early allograft dysfunction; 10.3% vs. 33.3%)
Kornberg A et al. [42]	2015	Prostaglandin E1 analogue (Alprostadil) given after liver transplantation for HCC	Retrospective study including 106 patients 59 treated with Alprostadil Significantly longer 5-year recurrence-free survival (85.7% vs. 63.1%) in Alprostadil group
Amador et al. [43]	2007	Ischemic preconditioning (IP) in liver donors	Randomized controlled trial 30 patients in each group (IP donors vs. standard donors) Lower postoperative AST activity, fewer reoperations, lower risk of PNF in IP group
Robertson et al. [44] (RIPCOLT Trial)	2017	Remote ischemic preconditioning (RIP) in liver recipients	Randomized controlled trial 20 patients in each group RIP achieved with lower limb ischemia No significant benefits were observed during early postoperative period (90-days)
Dutkowski et al. [45]	2015	Machine perfusion using HOPE technique vs. static cold storage (SCS) in DCD liver transplantation	Retrospective study 25 HOPE vs. 50 SCS patients HOPE perfusion associated with better liver function, less ischemic cholangiopathy and biliary complications 1-year survival better after HOPE (90% vs. 69%)
Nasralla et al. [46]	2015	Normothermic machine perfusion (NMP) in DBD and DCD vs. SCS	Randomized controlled trial 220 patients NMP associated with better liver function and decreased risk of EAD 1-year graft and patient survival similar in both groups
Savier et al. [47]	2020	Controlled DCD liver transplantation with regional NMP vs. standard DBD	Multicenter retrospective study 100 DBD and 50 regional NMP patients Aminotransferase activity lower in postoperative period in NMP group EAD, biliary complications, 2-year graft and patient survival comparable in both groups

## Data Availability

Not applicable.

## References

[1] Pinna A.D., Yang T., Mazzaferro V., De Carlis L., Zhou J., Roayaie S., Shen F., Sposito C., Cescon M., Di Sandro S. (2018). Liver Transplantation and Hepatic Resection can Achieve Cure for Hepatocellular Carcinoma. Ann. Surg..

[2] Antoine C., Jasseron C., Dondero F., Savier E. (2020). The French national steering committee of donors after circulatory death Liver Transplantation from Controlled Donors After Circulatory Death Using Normothermic Regional Perfusion: An Initial French Experience. Liver Transplant..

[3] Zhao H., Alam A., Soo A.P., George A., Ma D. (2018). Ischemia-Reperfusion Injury Reduces Long Term Renal Graft Survival: Mechanism and Beyond. EBioMedicine.

[4] Chen Z., Zhang P., Xu Y., Yan J., Liu Z., Lau W.B., Lau B., Li Y., Zhao X., Wei Y. (2019). Surgical stress and cancer progression: The twisted tango. Mol. Cancer.

[5] Van Der Bilt J.D.W., Kranenburg O., Nijkamp M.W., Smakman N., Veenendaal L.M., Velde E.A.T., Voest E.E., Van Diest P.J., Rinkes I.H.M.B. (2005). Ischemia/reperfusion accelerates the outgrowth of hepatic micrometastases in a highly standardized murine model. Hepatology.

[6] Grąt M., Krawczyk M., Wronka K.M., Stypułkowski J., Lewandowski Z., Wasilewicz M., Krawczyk P., Grąt K., Patkowski W., Zieniewicz K. (2018). Ischemia-reperfusion injury and the risk of hepatocellular carcinoma recurrence after deceased donor liver transplantation. Sci. Rep..

[7] Cameron A.M., Ghobrial R.M., Yersiz H., Farmer D.G., Lipshutz G.S., Gordon S.A., Zimmerman M., Hong J., Collins T.E., Gornbein J. (2006). Optimal Utilization of Donor Grafts with Extended Criteria: A single-center experience in over 1000 liver transplants. Ann. Surg..

[8] Wu M.-Y., Yiang G.-T., Liao W.-T., Tsai A., Cheng Y.-L., Cheng P.-W., Li C.-Y. (2018). Current Mechanistic Concepts in Ischemia and Reperfusion Injury. Cell. Physiol. Biochem..

[9] Granger D.N., Kvietys P.R. (2015). Reperfusion injury and reactive oxygen species: The evolution of a concept. Redox Biol..

[10] Cursio R., Colosetti P., Gugenheim J. (2015). Autophagy and Liver Ischemia-Reperfusion Injury. BioMed Res. Int..

[11] Baidya R., Crawford D.H.G., Gautheron J., Wang H., Bridle K.R. (2020). Necroptosis in Hepatosteatotic Ischaemia-Reperfusion Injury. Int. J. Mol. Sci..

[12] Konishi T., Lentsch A.B. (2017). Hepatic Ischemia/Reperfusion: Mechanisms of Tissue Injury, Repair, and Regeneration. Gene Expr..

[13] Nakamura K., Kageyama S., Kupiec-Weglinski J.W. (2019). The Evolving Role of Neutrophils in Liver Transplant Ischemia-Reperfusion Injury. Curr. Transplant. Rep..

[14] Perry B.C., Soltys D., Toledo A.H., Toledo-Pereyra L.H. (2011). Tumor Necrosis Factor-α in Liver Ischemia/Reperfusion Injury. J. Investig. Surg..

[15] Peralta C., Jiménez-Castro M.B., Gracia-Sancho J. (2013). Hepatic ischemia and reperfusion injury: Effects on the liver sinusoidal milieu. J. Hepatol..

[16] Datta G. (2013). Molecular mechanisms of liver ischemia reperfusion injury: Insights from transgenic knockout models. World J. Gastroenterol..

[17] Jiménez-Castro M.B., Cornide-Petronio M.E., Gracia-Sancho J., Peralta C. (2019). Inflammasome-Mediated Inflammation in Liver Ischemia-Reperfusion Injury. Cells.

[18] Dar W.A., Sullivan E., Bynon J.S., Eltzschig H., Ju C. (2019). Ischaemia reperfusion injury in liver transplantation: Cellular and molecular mechanisms. Liver Int..

[19] Huang S., Ju W., Zhu Z., Han M., Sun C., Tang Y., Hou Y., Zhang Z., Yang J., Zhang Y. (2019). Comprehensive and combined omics analysis reveals factors of ischemia-reperfusion injury in liver transplantation. Epigenomics.

[20] Kan C., Ungelenk L., Lupp A., Dirsch O., Dahmen U. (2018). Ischemia-Reperfusion Injury in Aged Livers—The Energy Metabolism, Inflammatory Response, and Autophagy. Transplantion.

[21] Blok J.J., Detry O., Putter H., Rogiers X., Porte R.J., Van Hoek B., Pirenne J., Metselaar H.J., Lerut J.P., Ysebaert D.K. (2016). Longterm results of liver transplantation from donation after circulatory death. Liver Transplant..

[22] Ling C.-C., Ng K.T.-P., Shao Y., Geng W., Xiao J.-W., Liu H., Li C.-X., Liu X.-B., Ma Y.-Y., Yeung W.-H. (2014). Post-transplant endothelial progenitor cell mobilization via CXCL10/CXCR3 signaling promotes liver tumor growth. J. Hepatol..

[23] Lim C., Broqueres-You D., Brouland J.P., Merkulova-Rainon T., Faussat A.-M., Hilal R., Rouquie D., Eveno C., Audollent R., Levy B.I. (2013). Hepatic ischemia-reperfusion increases circulating bone marrow-derived progenitor cells and tumor growth in a mouse model of colorectal liver metastases. J. Surg. Res..

[24] Li C.X., Ling C.C., Shao Y., Xu A., Li X.C., Ng K.T.-P., Liu X.B., Ma Y.Y., Qi X., Liu H. (2016). CXCL10/CXCR3 signaling mobilized-regulatory T cells promote liver tumor recurrence after transplantation. J. Hepatol..

[25] Van Der Bilt J.D., Soeters M.E., Duyverman A.M., Nijkamp M.W., Witteveen P.O., Van Diest P.J., Kranenburg O., Rinkes I.H.B. (2007). Perinecrotic Hypoxia Contributes to Ischemia/Reperfusion-Accelerated Outgrowth of Colorectal Micrometastases. Am. J. Pathol..

[26] Tamagawa K., Horiuchi T., Uchinami M., Doi K., Yoshida M., Nakamura T., Sasaki H., Taniguchi M., Tanaka K. (2008). Hepatic Ischemia-Reperfusion Increases Vascular Endothelial Growth Factor and Cancer Growth in Rats. J. Surg. Res..

[27] Nicoud I.B., Jones C.M., Pierce J.M., Earl T.M., Matrisian L.M., Chari R.S., Gorden D.L. (2007). Warm Hepatic Ischemia-Reperfusion Promotes Growth of Colorectal Carcinoma Micrometastases in Mouse Liver via Matrix Metalloproteinase-9 Induction. Cancer Res..

[28] Egeblad M., Werb Z. (2002). New functions for the matrix metalloproteinases in cancer progression. Nat. Rev. Cancer.

[29] Hamaguchi Y., Mori A., Fujimoto Y., Ito T., Iida T., Yagi S., Okajima H., Kaido T., Uemoto S. (2016). Longer warm ischemia can accelerate tumor growth through the induction of HIF-1α and the IL-6-JAK-STAT3 signaling pathway in a rat hepatocellular carcinoma model. J. Hepato Biliary Pancreat. Sci..

[30] Man K., Lo C.M., Xiao J.W., Ng K.T.-P., Sun B.S., Ng I.O.-L., Cheng Q., Sun C.K., Fan S.T. (2008). The Significance of Acute Phase Small-for-Size Graft Injury on Tumor Growth and Invasiveness after Liver Transplantation. Ann. Surg..

[31] Orci L.A., Lacotte S., Oldani G., Slits F., De Vito C., Crowe L.A., Rubbia-Brandt L., Vallée J., Morel P., Toso C. (2016). Effect of ischaemic preconditioning on recurrence of hepatocellular carcinoma in an experimental model of liver steatosis. BJS.

[32] Lee K.F., Chong C.C.N., Cheung S.Y.S., Wong J., Fung A.K.Y., Lok H.T., Lai P.B.S. (2019). Impact of Intermittent Pringle Maneuver on Long-Term Survival After Hepatectomy for Hepatocellular Carcinoma: Result from Two Combined Randomized Controlled Trials. World J. Surg..

[33] Xia F., Lau W.-Y., Xu Y., Wu L., Qian C., Bie P. (2012). Does Hepatic Ischemia–Reperfusion Injury Induced by Hepatic Pedicle Clamping Affect Survival after Partial Hepatectomy for Hepatocellular Carcinoma?. World J. Surg..

[34] Kornberg A., Witt U., Kornberg J., Friess H., Thrum K. (2015). Extended Ischemia Times Promote Risk of HCC Recurrence in Liver Transplant Patients. Dig. Dis. Sci..

[35] Nagai S., Yoshida A., Facciuto M., Moonka D., Abouljoud M.S., Schwartz M.E., Florman S.S. (2015). Ischemia time impacts recurrence of hepatocellular carcinoma after liver transplantation. Hepatology.

[36] Orci L.A., Berney T., Majno P.E., Lacotte S., Oldani G., Morel P., Mentha G., Toso C. (2015). Donor characteristics and risk of hepatocellular carcinoma recurrence after liver transplantation. BJS.

[37] Salgia R.J., Goodrich N.P., Marrero J.A., Volk M.L. (2013). Donor Factors Similarly Impact Survival Outcome after Liver Transplantation in Hepatocellular Carcinoma and Non-hepatocellular Carcinoma Patients. Dig. Dis. Sci..

[38] Khorsandi S.E., Yip V., Cortes M., Jassem W., Quaglia A., O’Grady J., Heneghan M., Aluvihare V., Agarwal K., Menon K. (2016). Does Donation After Cardiac Death Utilization Adversely Affect Hepatocellular Cancer Survival?. Transplantation.

[39] Silverstein J., Roll G., Dodge J.L., Grab J.D., Yao F.Y., Mehta N. (2020). Donation After Circulatory Death Is Associated With Similar Posttransplant Survival in All but the Highest-Risk Hepatocellular Carcinoma Patients. Liver Transplant..

[40] Santiago F., Bueno P., Olmedo C., Muffak-Granero K., Comino A., Serradilla M., Mansilla A., Villar J., Garrote D., Ferron J. (2008). Effect of N-Acetylcysteine Administration on Intraoperative Plasma Levels of Interleukin-4 and Interleukin-10 in Liver Transplant Recipients. Transplant. Proc..

[41] Ito T., Nakamura K., Kageyama S., Korayem I.M., Hirao H., Kadono K., Aziz J., Younan S., DiNorcia J., Agopian V.G. (2019). Impact of Rifaximin Therapy on Ischemia/Reperfusion Injury in Liver Transplantation: A Propensity Score–Matched Analysis. Liver Transplant..

[42] Kornberg J., Witt U., Friess H., Thrum K. (2015). Treating ischaemia-reperfusion injury with prostaglandin E1 reduces the risk of early hepatocellular carcinoma recurrence following liver transplantation. Aliment. Pharmacol. Ther..

[43] Cescon M., Grazi G.L., Grassi A., Ravaioli M., Vetrone G., Ercolani G., Varotti G., D’Errico A., Ballardini G., Pinna A.D. (2006). Effect of ischemic preconditioning in whole liver transplantation from deceased donors. A pilot study. Liver Transplant..

[44] Robertson F.P., Goswami R., Wright G., Imber C., Sharma D., Malago M., Fuller B.J., Davidson B.R. (2017). Remote ischaemic preconditioning in orthotopic liver transplantation (RIPCOLT trial): A pilot randomized controlled feasibility study. HPB.

[45] Dutkowski P., Polak W.G., Muiesan P., Schlegel A., Verhoeven C.J., Scalera I., DeOliveira M.L., Kron P., Clavien P.-A. (2015). First Comparison of Hypothermic Oxygenated PErfusion Versus Static Cold Storage of Human Donation After Cardiac Death Liver Transplants. Ann. Surg..

[46] Nasralla D., Consortium for Organ Preservation in Europe, Coussios C.C., Mergental H., Akhtar M.Z., Butler A.J., Ceresa C.D.L., Chiocchia V., Dutton S.J., García-Valdecasas J.C. (2018). A randomized trial of normothermic preservation in liver transplantation. Nat. Cell Biol..

[47] Savier E., Lim C., Rayar M., Orlando F., Boudjema K., Mohkam K., Lesurtel M., Mabrut J.Y., Pittau G., Begdadi N. (2020). Favorable outcomes of liver transplantation from controlled circulatory death donors using normothermic regional perfusion compared to brain death donors. Transplantation.

[48] Xu Z., Yu J., Wu J., Qi F., Wang H., Wang Z., Wang Z. (2016). The Effects of Two Anesthetics, Propofol and Sevoflurane, on Liver Ischemia/Reperfusion Injury. Cell. Physiol. Biochem..

[49] Hsiao H.-T., Wu H., Huang P.-C., Tsai Y.-C., Liu Y.-C. (2016). The effect of propofol and sevoflurane on antioxidants and proinflammatory cytokines in a porcine ischemia–reperfusion model. Acta Anaesthesiol. Taiwanica.

[50] Zhao G., Shen X., Nan H., Yan L., Zhao H., Yu J., Lv Y. (2013). Remifentanil protects liver against ischemia/reperfusion injury through activation of anti-apoptotic pathways. J. Surg. Res..

[51] Lian Y.-H., Fang J., Zhou H.-D., Jiang H.-F., Xie K.-J. (2019). Sufentanil Preconditioning Protects Against Hepatic Ischemia-Reperfusion Injury by Suppressing Inflammation. Med. Sci. Monit..

[52] Abe T. (2004). A new free radical scavenger, edaravone, ameliorates oxidative liver damage due to ischemia-reperfusion in vitro and in vivo. J. Gastrointest. Surg..

[53] Nakamura A., Akamatsu Y., Miyagi S., Fukumori T., Sekiguchi S., Satomi S. (2008). A Free Radical Scavenger, Edaravone, Prevents Ischemia-Reperfusion Injury in Liver Grafts From Non–Heart-Beating Donors. Transplant. Proc..

[54] Beck-Schimmer B., Breitenstein S., Urech S., De Conno E., Wittlinger M., Puhan M., Jochum W., Spahn D.R., Graf R., Clavien P.-A. (2008). A Randomized Controlled Trial on Pharmacological Preconditioning in Liver Surgery Using a Volatile Anesthetic. Ann. Surg..

[55] Beck-Schimmer B., Breitenstein S., Bonvini J.M., Lesurtel M., Ganter M., Weber A., Puhan M.A., Clavien P.-A. (2012). Protection of Pharmacological Postconditioning in Liver Surgery: Results of a prospective randomized controlled trial. Ann. Surg..

[56] Abu-Amara M., Gurusamy K., Hori S., Glantzounis G., Fuller B., Davidson B.R. (2010). Systematic review of randomized controlled trials of pharmacological interventions to reduce ischaemia-reperfusion injury in elective liver resection with vascular occlusion. HPB.

[57] Glantzounis G.K., Yang W., Koti R.S., Mikhailidis D.P., Seifalian A., Davidson B.R. (2004). Continuous infusion of N-acetylcysteine reduces liver warm ischaemia–reperfusion injury. BJS.

[58] Grąt M., Wronka K.M., Lewandowski Z., Grąt K., Krasnodębski M., Stypułkowski J., Hołówko W., Masior Ł., Kosińska I., Wasilewicz M. (2017). Effects of continuous use of probiotics before liver transplantation: A randomized, double-blind, placebo-controlled trial. Clin. Nutr..

[59] Rodríguez-Reynoso S., Leal-Cortés C., Buen E.P.-D., La Torre S.P.L.-D. (2018). Ischemic Preconditioning Preserves Liver Energy Charge and Function on Hepatic Ischemia/Reperfusion Injury in Rats. Arch. Med. Res..

[60] Choi E.K., Jung H., Jeon S., Lim J.A., Lee J., Kim H., Hong S.W., Jang M.H., Lim D.G., Kwak K.H. (2020). Role of Remote Ischemic Preconditioning in Hepatic Ischemic Reperfusion Injury. Dose Response.

[61] Li D.-Y., Shi X.-J., Li W., Sun X.-D., Wang G.-Y. (2016). Ischemic preconditioning and remote ischemic preconditioning provide combined protective effect against ischemia/reperfusion injury. Life Sci..

[62] Oldani G., Crowe L.A., Orci L.A., Slits F., Rubbia-Brandt L., de Vito C., Morel P., Mentha G., Berney T., Vallée J.-P. (2014). Pre-retrieval reperfusion decreases cancer recurrence after rat ischemic liver graft transplantation. J. Hepatol..

[63] Amador A., Grande L., Marti J., Deulofeu R., Miquel R., Solá A., Laiz G.R., Ferrer J., Fondevila C., Charco R. (2007). Ischemic Pre-conditioning in Deceased Donor Liver Transplantation: A Prospective Randomized Clinical Trial. Arab. Archaeol. Epigr..

[64] Koneru B., Shareef A., Dikdan G., Desai K., Klein K.M., Peng B., Wachsberg R.H., De La Torre A.N., Debroy M., Fisher A. (2007). The Ischemic Preconditioning Paradox in Deceased Donor Liver Transplantation—Evidence from a Prospective Randomized Single Blind Clinical Trial. Arab. Archaeol. Epigr..

[65] Robertson F.P., Magill L.J., Wright G.P., Fuller B., Davidson B.R. (2016). A systematic review and meta-analysis of donor ischaemic preconditioning in liver transplantation. Transpl. Int..

[66] Jung K.-W., Kang J., Kwon H.-M., Moon Y.-J., Jun I.-G., Song J.-G., Hwang G.-S. (2020). Effect of Remote Ischemic Preconditioning Conducted in Living Liver Donors on Postoperative Liver Function in Donors and Recipients Following Liver Transplantation. Ann. Surg..

[67] Karangwa S.A., Dutkowski P., Fontes P., Friend P.J., Guarrera J.V., Markmann J.F., Mergental H., Minor T., Quintini C., Selzner M. (2016). Machine Perfusion of Donor Livers for Transplantation: A Proposal for Standardized Nomenclature and Reporting Guidelines. Arab. Archaeol. Epigr..

[68] Vogel T., Brockmann J.G., Quaglia A., Morovat A., Jassem W., Heaton N.D., Coussios C., Friend P.J. (2016). The 24-hour normothermic machine perfusion of discarded human liver grafts. Liver Transplant..

[69] Eshmuminov D., Becker D., Borrego L.B., Hefti M., Schuler M.J., Hagedorn C., Muller X., Mueller M., Onder C., Graf R. (2020). An integrated perfusion machine preserves injured human livers for 1 week. Nat. Biotechnol..

[70] Schlegel A., Graf R., Clavien P.-A., Dutkowski P. (2013). Hypothermic oxygenated perfusion (HOPE) protects from biliary injury in a rodent model of DCD liver transplantation. J. Hepatol..

[71] Nassar A., Liu Q., Farias K., D’Amico G., Tom C., Grady P., Bennett A., Uso T.D., Eghtesad B., Kelly D. (2014). Ex Vivo Normothermic Machine Perfusion Is Safe, Simple, and Reliable. Surg. Innov..

[72] Oldani G., Peloso A., Slits F., Gex Q., Delaune V., Orci L.A., Van De Looij Y., Colin D.J., Germain S., De Vito C. (2019). The impact of short-term machine perfusion on the risk of cancer recurrence after rat liver transplantation with donors after circulatory death. PLoS ONE.

[73] Schlegel A., Muller X., Kalisvaart M., Muellhaupt B., Perera M.T.P., Isaac J.R., Clavien P.-A., Muiesan P., Dutkowski P. (2019). Outcomes of DCD liver transplantation using organs treated by hypothermic oxygenated perfusion before implantation. J. Hepatol..

[74] Mueller M., Kalisvaart M., O‘Rourke J., Shetty S., Parente A., Muller X., Isaac J., Muellhaupt B., Muiesan P., Shah T. (2020). Hypothermic Oxygenated Liver Perfusion (HOPE) Prevents Tumor Recurrence in Liver Transplantation From Donation After Circulatory Death. Ann. Surg..

[75] Van Rijn R., Karimian N., Matton A.P.M., Burlage L.C., Westerkamp A.C., Berg A.P.V.D., De Kleine R.H.J., De Boer M.T., Lisman T., Porte R.J. (2017). Dual hypothermic oxygenated machine perfusion in liver transplants donated after circulatory death. BJS.

[76] van Leeuwen O., De Vries Y., Fujiyoshi M., Nijsten M.W.N., Ubbink R., Pelgrim G.J., Werner M.J.M., Reyntjens K.M.E.M., Berg A.P.V.D., De Boer M.T. (2019). Transplantation of High-risk Donor Livers After Ex Situ Resuscitation and Assessment Using Combined Hypo- and Normothermic Machine Perfusion. Ann. Surg..

[77] Hessheimer A.J., Coll E., Torres F., Ruíz P., Gastaca M., Rivas J.I., Gómez M., Sánchez B., Santoyo J., Ramírez P. (2019). Normothermic regional perfusion vs. super-rapid recovery in controlled donation after circulatory death liver transplantation. J. Hepatol..

[78] Yazdani H.O., Kaltenmeier C., Morder K., Moon J., Traczek M., Loughran P., Zamora R., Vodovotz Y., Li F., Wang J.H. (2020). Exercise Training Decreases Hepatic Injury and Metastases Through Changes in Immune Response to Liver Ischemia/Reperfusion in Mice. Hepatology.

[79] Ito T., Naini B.V., Markovic D., Aziz A., Younan S., Lu M., Hirao H., Kadono K., Kojima H., DiNorcia J. (2020). Ischemia-reperfusion injury and its relationship with early allograft dysfunction in liver transplant patients. Arab. Archaeol. Epigr..

[80] Bastos-Neves D., Salvalaggio P.R.D.O., De Almeida M.D. (2019). Risk factors, surgical complications and graft survival in liver transplant recipients with early allograft dysfunction. Hepatobiliary Pancreat. Dis. Int..

[81] He X., Guo Z., Zhao Q., Ju W., Wang D., Wu L., Yang L., Ji F., Tang Y., Zhang Z. (2017). The first case of ischemia-free organ transplantation in humans: A proof of concept. Arab. Archaeol. Epigr..

